# Fertility preservation in endometrial cancer patients: options, challenges and perspectives

**DOI:** 10.3332/ecancer.2020.1030

**Published:** 2020-05-06

**Authors:** Milan Terzic, Melanie Norton, Sanja Terzic, Gauri Bapayeva, Gulzhanat Aimagambetova

**Affiliations:** 1 Clinical Academic Department of Women’s Health, National Research Center for Mother and Child Health, University Medical Center, Astana, Kazakhstan; 2 Department of Medicine, Nazarbayev University, School of Medicine, Astana, Kazakhstan; 3 Department of Obstetrics, Gynecology and Reproductive Sciences, University of Pittsburgh, School of Medicine, Pittsburgh, Pennsylvania, USA; 4 Whittington Hospital, Department of Urogynaecology, Magdala Ave, London N19 5NF, UK; 5 Department of Biomedical Sciences, Nazarbayev University, School of Medicine, Astana, Kazakhstan; a http://orcid.org/0000-0003-3914-5154; b http://orcid.org/0000-0002-2868-4497

**Keywords:** endometrial cancer, fertility preservation, conservative management intrauterine system, IUD, hysteroscopy

## Abstract

Several different approaches have been designed by physicians in order to preserve fertility in younger patients with endometrial carcinoma. There are various options offering different advantages, but hysteroscopic resection of pathologic endometrial tissue with placement of a Levonorgestrel Intrauterine Device has proven to be the most successful in allowing patients to conceive and give birth afterwards. However, conservative treatments should only be considered in patients with low-grade and low-stage endometrial tumours. There are many published studies which have sought out a preferable approach to treating endometrial cancer whilst preserving fertility. However, more research on this matter is needed to allow a better understanding as to which techniques/approaches are optimal. In this review, we will summarise the current available treatment options for endometrial cancer in patients of reproductive age.

## Introduction

Endometrial cancer (EC), also known as adenocarcinoma of the endometrium, is the most common malignancy of the female genital tract in developed countries [1, 2]. In the United States, EC incidence was 61,380 new uterine cancer cases in 2017, with 10,920 deaths resulting from the disease [1, 2]. In the epidemiological study by Lortet-Tieulent *et al* [3], the EC incidence rate was found to increase over time in several countries. The highest rates were in North America, Eastern and Northern Europe (19 cases per 100,000 among whites in the United States, 95% confidence interval [CI] = 18 to 20, and in Slovakia, 95% CI = 18 to 21), and the lowest rates were in middle-income countries (South Africa 1, 95% CI = 0 to 3, and India 3, 95% CI = 3 to 4) [3].

Although rare in young patients, cancer of the endometrium can affect reproductive age women (12–51 years) [4]. It has been estimated that worldwide each year, EC develops in about 142,000 women [5]. Despite an overall 5-year survival of 80%, the estimated annual mortality rate is 42,000 [6]. The majority of cases are diagnosed in post-menopausal patients averaging around 75 years of age [5, 7]. However, women of reproductive age (as young as 12) can also develop this dangerous type of cancer [7].

The etiology and pathogenesis of EC have not yet been fully understood. Currently, epigenetic modifications were reported to be useful in the explanation of disease-specific features. For a better understanding of EC etiology, MiRNAs that act as part of the epigenetic machinery should be studied. It is known that TrkB-STAT3-miR-204-5p regulatory circuitry controls endometrial carcinoma cells [8]. Dysregulation of miRNA-204 mediates migration and invasion of endometrial carcinoma. Researchers identified suppression of miR-204-5p in endometrial carcinoma compared to miRNA expression in normal tissues based on validation from the Cancer Genome Atlas (TCGA) dataset [8]. MiR-204-5p expression was found to be lower in the endometrial carcinoma tissues than in adjacent normal tissues from TCGA.

Risk factors for uterine neoplasms include early menarche, nulliparity, late age at menopause, increased levels of estrogen caused by obesity, diabetes, high-fat diet, advanced age (≥55 years) and tamoxifen use [1]. Therefore, the growing incidence of EC could be attributed to increased life expectancy and obesity. Most EC is caused by sporadic mutations. However, genetic mutations could cause EC in about 5% of patients, which occurs 10 to 20 years before sporadic cancer [1, 9].

Postmenopausal bleeding is the main characteristic symptom that leads to investigations and early-stage diagnosis [10]. This is easily recognised in women that have already gone through menopause, however, this irregular bleeding can remain difficult to identify in younger women who are still menstruating regularly. The investigations that help with a diagnosis are transvaginal ultrasound measuring of endometrial thickness, hysteroscopy [11–13] and pathohistological analysis of endometrial biopsies [14, 15].

Adenocarcinomas make up the majority of EC [16]. They are derived from the epithelial cells that line the endometrium [17]. They can be classified into two types (Type 1 and Type 2) [18]. Type 1 endometrial adenocarcinomas are more common and tend to develop in a younger cohort of women including those that are perimenopausal [19]. They are often estrogen-dependent, low-grade and minimally invasive which is the reason that they are linked to a better prognosis. They are usually seen in cases of prolonged unopposed estrogen exposure [20].

Type 2 endometrial carcinomas tend to surface in a slightly older population that is postmenopausal [19]. They are not estrogen-dependent, typically aggressive, high grade and deeply invade the myometrium, hence having a poorer prognosis [21]. Analysing histological characteristics of the endometrial tumour helps to determine the prognosis.

Currently, the abovementioned traditional view of the pathogenesis of EC is changing after the molecular studies by TCGA, which issued an integrated report of genomic, transcriptomic and proteomic profiles in 373 patients diagnosed with EC [22]. Molecular data have been used to further stratify risk categories. The researchers determined four prognostic categories for classification of EC: (1) polymerase ɛ ultramutated, (2) microsatellite instability hypermutated, (3) low copy number and (4) high copy number [23].

Several new agents are under evaluation for treating patients with metastatic, recurrent and persistent EC. Molecular characterisation has also been pursued potential therapeutic targets in EC, focusing on frequently mutated pathways, such as PI3K/PTEN/AKT/mTOR. According to expert opinion, in the near future, new studies with dual inhibitors or multi-pathways inhibitors as mono or combination therapies with conventional chemotherapy or other targeted drugs may provide more promising data. Moreover, the evaluation of new serum and histological biomarkers is an attractive strategy for patient selection [24].

Optimal management of patients with complex atypical hyperplasia or EC who desire future fertility is unknown [25]. However, some conservative methods have been introduced in order to preserve female fertility [26–28]. The aim of our review is to summarise heterogeneous literature sources and makes the data more understandable.

## Minimally invasive interventions

After proper surgical work-up [29], surgical removal of the uterus with the fallopian tubes and ovaries is the main treatment of choice. Over the past years, clinical practice develops in a direction to minimally invasive approaches to total hysterectomy with bilateral salpingo-oophorectomy (TH/BSO) and lymph node assessment, especially in young patients in patients with early-stage EC [1, 30]. Although these procedures may be performed by any surgical route (laparoscopic, robotic, vaginal and abdominal), the standard in those with the apparent uterine-confined disease is to perform the procedure via a minimally invasive approach [1, 31]. There are still debates as to which type of procedure is deemed most effective [32].

Efficacy of the minimally invasive interventions was justified in a study that compared laparoscopy versus laparotomy approach to the treatment of EC among 2,616 patients with clinical stage I to IIA of the disease [1, 33, 34]. Interestingly enough, fewer postoperative adverse events and shorter stay in hospital occurred with laparoscopy compared with laparotomy. Recurrence rates were 11.4% for laparoscopy versus 10.2% for laparotomy. The 5-year overall survival rate was 84.8% [1, 34]. However, another study results compared the outcomes of patients with stage I endometrial carcinoma (*n* = 760), who randomly underwent an abdominal or laparoscopic hysterectomy, reveals no significant differences (at a median follow-up of 4.5 years, disease-free survival was 81.3% for laparotomy versus 81.6% for laparoscopy) [35].

Regarding the robotic surgical approach, a review of previously published data on the efficacy of laparoscopic versus robotic surgery for hysterectomies suggests that robotic hysterectomies (RHs) take longer but may be associated with a shorter hospital stay [36]. On the other hand, in recent meta-analysis, researchers show that RH in EC may have advantages in reducing overall complications, length of hospital admission, estimated blood loss, transfusion and readmission compared to those for open hysterectomy and laparoscopic hysterectomy [36, 37]. Therefore, RH may be a generally safer and better option than for patients with EC.

Furthermore, in the last decades, the evaluation of sentinel lymph node utilising a robotic platform for EC staging has gained evidence among gynaecologic oncologists in parallel with the important role of minimally invasive surgical approaches [38]. This approach will decrease the number of lymphadenectomies for low-risk early-stage EC.

Depending on the tumour staging, radiotherapy and chemotherapy may also be necessary for the treatment of EC [39, 40].

## Fertility-sparing therapy

Although the primary treatment of EC is usually a hysterectomy, continuous progestin-based therapy may be considered for a selected group of young patients with Grade 1, stage IA (non-invasive) disease who wish to preserve their fertility [1, 41, 42]. It may also be used for young patients with endometrial hyperplasia who desire fertility preservation.

While considering fertility-sparing therapy, all of the following criteria must be met [1]:

1. Well-differentiated (grade 1) endometrioid adenocarcinoma on dilation and curettage (D&C) confirmed by expert pathology review
2. Disease limited to the endometrium on MRI (preferred) or transvaginal ultrasound
3. Absence of suspicious or metastatic disease on imaging
4. No contraindications to medical therapy or pregnancy
5. Patients should undergo counselling that fertility-sparing option is NOT standard of care for the treatment of endometrial carcinoma

Selected patients, who will meet all the criteria, may require genetic counselling and testing. Furthermore, TH/BSO with surgical staging is recommended after childbearing is complete, if therapy is not effective, or if progression occurs. Fertility-sparing therapy is not recommended for patients with high-grade endometrioid adenocarcinomas, uterine serous carcinoma, clear cell carcinoma and carcinosarcoma [1].

### Progestins (high/low dose)

According to the published literature, progestin tends to be the most frequent type of conservative treatment of early-stage EC [43]. In the 90’s, it was first described as a successful hormonal treatment that allowed patients with EC to become pregnant [44, 45]. Since then, it has become increasingly more popular in terms of non-invasive treatments.

Continuous progestin therapy may include megestrol acetate (MA), medroxyprogesterone acetate (MPA) or an intrauterine device containing levonorgestrel [1, 46–48]. To this day, there is still no definitive answer as to what is the optimal dose and duration of treatment that produces the best effects. However, the most common reported doses to vary between 250 and 600 mg/day for MPA and 160–480 mg/day for MA [49].

A complete response of treatment occurs in about 50% of patients on average around 5.5 months of continuous use [1, 47, 49]. Despite a positive response to progestin use, several cases have reported a recurrence rate in about one-fifth of patients taking progestins [47]. Recurrence was reported to take place after an average of 23+/− months [49]. Relapse also did occur in a very small percentage of patients after they initially reached a complete response to conservative progestin treatment [47].

Unfortunately, progestin supplementation has its shortcomings [50, 51]. Long-term oral administration of progesterone can cause certain adverse effects in patients including abdominal cramps, depression, dizziness and headaches [52]. For this reason, complete compliance has proven to be quite difficult to achieve from patients in many studies [53–55]. Therefore, other conservative approaches have been researched, including levonorgestrel-releasing intrauterine devices and the use of gonadotropin-releasing hormones. These may either be used separately or together.

Another study combined MA with tamoxifen and gonadotropin-releasing hormone analogue (GnRHa) and found that 89% of patients reached complete remission after therapy with this combination of hormones [56]. Furthermore, 45% of patients were able to successfully conceive after the treatment. The prognosis was good in the majority of these patients [56].

In patients receiving progestin-based therapies, the NCCN panel recommends close monitoring with endometrial sampling every 3 to 6 months [1]. TH/BSO with staging is recommended (1) after childbearing is complete, (2) if patients have documented progression with biopsies and (3) if EC is still present after 6 to 12 months of progestin-based therapy [1, 42, 57].

The use of progestin-based therapy should be administered carefully by taking into consideration the contraindications: breast cancer, stroke, myocardial infarction, pulmonary embolism, deep vein thrombosis and smoking [1].

### Levonorgestrel-releasing intrauterine device

In recent years, more and more patients accepted a levonorgestrel-releasing intrauterine device (LNG-IUD) as an option for conservative management of endometrial hyperplasia and EC [58, 59]. It is already well known that LNG-IUD can provoke a higher progesterone concentration than oral progestin. However, results of small studies which are used LNG-IUD for EC have inconclusive results [25].

One study solely focused on patients with stage 1 grade 1 EC with positive progesterone receptors. All patients were assessed by anaesthesiologists who deemed them unfit for surgery with general anaesthesia [60]. Out of four cases, only one patient (25%) successfully had tumours regression in response to the LNG-IUD However, several cases have been reported that the LNG-IUD was not completely successful in preventing atypical endometrial hyperplasia from progressing to an adenocarcinoma. This raises questions about the efficacy of this approach.

In a more recent study of 46 patients, diagnosed with complex atypical hyperplasia or early grade EC, who were treated with the LNG-IUD, resulted in a return to normal histology in a majority of patients—the overall response rate was 75% (95% CI = 57–89) at 6 months [25].

A report from the recent meta-analysis states that for patients with EC/atypical hyperplasia, treatments with progestin, with or without LNG-IUD, or LNG-IUD alone can reach a good complete response rate; however, the pregnancy outcomes might be worse in patients treated with LNG-IUD alone [2]. Therefore, conservative treatments should be approached with caution and further randomised-controlled studies are necessary to confirm and update these data.

### Progestin use with levonorgestrel-releasing intrauterine device

One Korean study tested the effectiveness of oral progestins (MPA) with the use of LNG-IUD [61]. Patients who were under 40 years, who desired to preserve their fertility and who had a low-grade EC were included in the study. All patients were fitted with an LNG-IUD and were supplemented to take 500 mg daily of MPA. The complete remission rate was 87.5% and this was achieved approximately 9.8 months after the start of treatment. Around 12% of patients continued to have successful pregnancies [61]. It seems that combined treatment is more successful than the use of an LNG-IUD by itself.

### Levonorgestrel intrauterine device with gonadotropin releasing hormone agonists

A meta-analysis helped shed some light on the utilisation of LNG-IUD in combination with gonadotropin-releasing hormone agonists (GnRHa). In this study, data from six investigations were summarised which comprised 90 patients with an early stage of EC [59]. A total of 75.5% (68/90) of patients achieved complete response (95% CI, 60.4%–82.5%). Among the 68 patients, 34 patients were prepared for pregnancy, and 20 patients got pregnant successfully, of which 13 patients conceived successfully via ART and 3 via natural method [58].

A Russian study found 72% of patients with early-stage endometrial adenocarcinoma who used LNG-IUD with GnRHa had reached complete remission [62]. Eight patients from the study developed 10 conceptions that later turned into 8 live births [62]. These positive results raise the question if the involvement of progestins in the conservative treatment of EC is necessary.

Another study with a similar design found 88% of women with EC who had GnRHa injections with an LNG-IUD had a complete response after about 18.7 months. These results are very promising [63].

Based on the abovementioned data, we can conclude that recent intrauterine progestin therapy, such as LNG-IUD combined with GnRHa, has a satisfactory pregnancy rate and low recurrence rate [59].

### Hysteroscopic resection followed by progestin therapy

The results of the recent meta-analysis show that patients who received hysteroscopic resection followed by progestin therapy achieved the highest complete response rate [59]. There were six similar studies analysed with a total number of 73 presumed stage IA EC patients who underwent hysteroscopic resection of the lesions, the endometrium adjacent to the carcinoma, and the myometrium underlying the tumour followed by progestin therapy. A total of 89% (65/73) of patients achieved a pathological complete response, with a pooled complete response of 95.3% (95% CI, 87.8%–100%).

The other study focused on the efficacy of surgical resection of the diseased endometrial followed by oral therapy of MA 160 mg/day commences 5 days after the procedure for a total of 6 months [64]. All patients were nulliparous at diagnosis of endometrial cancer. More than 65% of patients successfully gave birth to five infants without any reproductive assistance. They were able to conceive on average 24 months after the end of therapy. These results are exceptionally promising, however, physicians must take great care while curetting all necessary areas of the tumour in order to ensure appropriate removal [64]. Further well-designed, randomised controlled trials are necessary to confirm and update these data.

### Hysteroscopic resection followed by insertion of levonorgestrel-releasing intrauterine device

Several studies have looked at the effect of physically resecting the pathologic tissue through hysteroscopy and then apply an LNG-IUD. They found that 7% of patients had a cancer recurrence, and 78% of patients with endometrial carcinoma showed a complete response [65]. Almost half (45%) of the patients that achieved a complete response, after removal of LNG-IUD were able to conceive naturally and give birth within 12 months of receiving treatment. The results and success ratios were quite similar to those of patients who receive progestins as a form of conservative treatment [65].

Recently, hysteroscopic resection of atypical hyperplasia and EC followed by oral or intrauterine-released progestins has been demonstrated to be an effective fertility-sparing treatment [66]. The researchers’ findings strongly suggest that hysteroscopic resection followed by the insertion of LNG-IUD might have a lower relapse rate than progestin therapies alone, with the similar response and pregnancy rates [66].

## Conclusion

Women of reproductive age experiencing endometrial cancer require conservative management and counselling for fertility-sparing options. The management of young women with endometrial cancer and atypical hyperplasia varies among clinicians. The use of progestins seems to offer very good results in treating early-stage endometrial cancer and is useful for fertility preservation. However, the optimal treatment is still debated and no consensus has been reached yet. Furthermore, fertility-sparing management should not be restricted to infertile women. Operative hysteroscopy should be the preferred endometrial sampling method because of its association with a higher remission rate. The guidance for managing conservatively young patients with endometrial cancer should be provided, but should never replace the individual approach for care and management as each patient has different characteristics as well as different needs and expectations.

## Conflicts of interest

The authors declare that they have no conflict of interests with respect to this paper.

## Funding declaration

The authors did not receive specific funding for this work.

## Authors’ contributions

MT formulated the review. MN, ST, GB and GA planned the review, conducted the literature search and wrote the initial draft. MT and GA performed the final review of the manuscript.
